# JNETS clinical practice guidelines for gastroenteropancreatic neuroendocrine neoplasms: diagnosis, treatment, and follow-up: a synopsis

**DOI:** 10.1007/s00535-021-01827-7

**Published:** 2021-09-29

**Authors:** Tetsuhide Ito, Toshihiko Masui, Izumi Komoto, Ryuichiro Doi, Robert Y. Osamura, Akihiro Sakurai, Masafumi Ikeda, Koji Takano, Hisato Igarashi, Akira Shimatsu, Kazuhiko Nakamura, Yuji Nakamoto, Susumu Hijioka, Koji Morita, Yuichi Ishikawa, Nobuyuki Ohike, Atsuko Kasajima, Ryoji Kushima, Motohiro Kojima, Hironobu Sasano, Satoshi Hirano, Nobumasa Mizuno, Taku Aoki, Takeshi Aoki, Takao Ohtsuka, Tomoyuki Okumura, Yasutoshi Kimura, Atsushi Kudo, Tsuyoshi Konishi, Ippei Matsumoto, Noritoshi Kobayashi, Nao Fujimori, Yoshitaka Honma, Chigusa Morizane, Shinya Uchino, Kiyomi Horiuchi, Masanori Yamasaki, Jun Matsubayashi, Yuichi Sato, Masau Sekiguchi, Shinichi Abe, Takuji Okusaka, Mitsuhiro Kida, Wataru Kimura, Masao Tanaka, Yoshiyuki Majima, Robert T. Jensen, Koichi Hirata, Masayuki Imamura, Shinji Uemoto

**Affiliations:** 1 Neuroendocrine Tumor Centre, Fukuoka Sanno Hospital, 3-6-45 Momochihama, Sawara-ku, Fukuoka, 814-0001 Japan; 2 Department of Gastroenterology, Graduate School of Medical Sciences, Internal University of Health and Welfare, 3-6-45 Momochihama, Sawara-ku, Fukuoka, 814-0001 Japan

**Keywords:** Clinical practice guideline, Gastroenteropancreatic neuroendocrine neoplasm, Japanese Neuroendocrine Tumor Society

## Abstract

Neuroendocrine neoplasms (NENs) are rare neoplasms that occur in various organs and present with diverse clinical manifestations. Pathological classification is important in the diagnosis of NENs. Treatment strategies must be selected according to the status of differentiation and malignancy by accurately determining whether the neoplasm is functioning or nonfunctioning, degree of disease progression, and presence of metastasis. The newly revised Clinical Practice Guidelines for Gastroenteropancreatic Neuroendocrine Neoplasms (GEP-NENs) comprises 5 chapters—diagnosis, pathology, surgical treatment, medical and multidisciplinary treatment, and multiple endocrine neoplasia type 1 (MEN1)/von Hippel–Lindau (VHL) disease—and includes 51 clinical questions and 19 columns. These guidelines aim to provide direction and practical clinical content for the management of GEP-NEN preferentially based on clinically useful reports. These revised guidelines also refer to the new concept of “neuroendocrine tumor” (NET) grade 3, which is based on the 2017 and 2019 WHO criteria; this includes health insurance coverage of somatostatin receptor scintigraphy for NEN, everolimus for lung and gastrointestinal NET, and lanreotide for GEP-NET. The guidelines also newly refer to the diagnosis, treatment, and surveillance of NEN associated with VHL disease and MEN1. The accuracy of these guidelines has been improved by examining and adopting new evidence obtained after the first edition was published.

**Supplementary Information:**

The online version contains supplementary material available at 10.1007/s00535-021-01827-7.

## Introduction

To standardize the diagnosis and treatment of gastroenteropancreatic neuroendocrine neoplasms (GEP-NENs) in Japan, the Japanese Neuroendocrine Tumor Society (JNETS) published the first Clinical Practice Guidelines for Gastroenteropancreatic Neuroendocrine Neoplasms in Japan in 2015 [1]. However, several subsequent developments regarding neuroendocrine neoplasms (NENs) necessitate the revision of clinical practice guidelines. The Guidelines Revision Committee was established at JNETS and began working on the revised guidelines in January 2018.

After making updates to reflect the assessment committee members’ suggestions, public hearings were held at various academic societies starting April 2019, culminating in the publication of the second edition in September 2019 [2]. This revised edition encompasses diagnosis, pathology, surgical treatment, medical and multidisciplinary treatment, and multiple endocrine neoplasia type 1 (MEN1)/von Hippel–Lindau (VHL) disease and includes 51 clinical questions and 19 columns. Topics under exploration are introduced in the “Columns” in the guideline based on expert consensus and evidence.

As a new development in the treatment of NEN in Japan, somatostatin receptor scintigraphy (SRS) [3, 4] was approved for insurance coverage in 2015 for the general diagnosis of NEN. In addition, the WHO classification for GEP-NENs was revised in 2017 and 2019, adding the new grade 3 (G3) well-differentiated neuroendocrine tumors (NETs), which are characterized by well-differentiated tissue and a Ki-67 index exceeding 20% [5, 6]. Regarding surgical treatment, the first edition of the guidelines did not include explicit recommendations for nonfunctioning pancreatic NETs 1–2 cm in size. However, the revised edition specifies indications for surgery and recommends surgical approaches for well-differentiated nonfunctioning pancreatic NETs and specifically covers the management of small pancreatic NETs from a broad perspective. The revised guidelines also newly include indications for surgery for poorly differentiated pancreatic neuroendocrine carcinomas (NECs). Regarding drug therapy, the molecular targeted drug everolimus is now covered by insurance for the treatment of well-differentiated NENs of the lungs and gastrointestinal tract [7]. In addition, the somatostatin analogue lanreotide is now covered by insurance for the treatment of well-differentiated GEP-NENs [8–10], substantially broadening treatment options. Moreover, while the first edition covered the diagnosis and treatment of NENs associated with MEN1, the revised edition also covers pancreatic NENs associated with VHL disease [11]. In this article, we explain the changes described above in the sequential order of the 5 chapters of the guidelines.

## Diagnosis

A GEP-NEN can be functioning or nonfunctioning. A Japanese epidemiological study reports that approximately 35% of pancreatic NETs are functioning, indicating that most pancreatic NETs are nonfunctioning [12]. Meanwhile, approximately 1% of gastrointestinal NETs present with carcinoid syndrome, which differs considerably from the trends in Europe and the U.S. [12]. This is likely because hindgut NENs are more prevalent in Japan, while midgut NENs, which have higher rates of carcinoid syndrome complication, are more prevalent in Europe and the U.S. [12, 13].

Functional NENs are often diagnosed on the basis of endocrine symptoms due to hormonal hypersecretion. Insulinoma primarily presents with fasting hypoglycemia episodes and includes autonomic and neurologic symptoms. In cases in which hypoglycemic symptoms are unrecognized, symptoms such as seizures and dementia may be the earliest symptoms [14, 15]. Testing such as a 72-h fasting test and a mixed-meal test are recommended for definitive diagnosis [16], although there are recent reports of a 48-h fasting test combined with a glucagon test [17]. Symptoms of gastrinoma include peptic ulcer and reflux esophagitis due to gastric hypersecretion and diarrhea due to pancreatic enzyme inactivation [18]. Measurement of fasting serum gastrin level and gastric acid pH are required for definitive diagnosis, while a calcium infusion test is useful [19, 20]. Determination of MEN1 complication is also recommended [21, 22]. Symptoms and recommended tests for functional NENs including other relatively rare neoplasms are shown in Table 1.

**Table 1:** Symptoms and recommended tests for functional NENs

Functional neuroendocrine neoplasm	Symptoms and findings	Differential diagnosis (presence diagnosis)
Insulinoma	Central nervous system symptoms: impaired consciousness (67–80%), abnormal vision (42–59%), amnesia (47%), personality changes (16–38%), epilepsy (16–17%), headache (7%) Autonomic symptoms: sweating (30–69%), malaise (28–56%), hyperphagia/obesity (14–50%), tremor (12–14%), palpitation (5–12%), anxiety (12%)	Differentiating hypoglycemia: Whipple’s triad, exogenous insulin, oral hypoglycemic agents, endogenous insulin dyssecretion, insulin autoimmune syndrome Definitive diagnosis: 72-h fasting test, mixed-meal test, 48-h fasting test + glucagon tolerance test
Gastrinoma	Peptic ulcers: duodenal bulb (75%), distal duodenum (14%), jejunum (11%) Abdominal pain, steatorrhea	Fasting serum gastrin measurement, gastric pH measurement, intravenous calcium injection test (MEN1 differential diagnostics: blood calcium measurement, intact PTH measurement)
Glucagonoma	Glucose intolerance/diabetes (30–90%), weight loss (60–90%), necrotizing erythema migrans (55–90%), mucosal symptoms (30–40%), diarrhea (10–15%), anemia (30–90%), hypoaminoacidemia (30–100%), venous thrombosis, psychoneurotic symptoms	Plasma glucagon measurement, serum albumin measurement, amino acid fraction measurement
VIPoma	Profuse watery diarrhea, hypokalemia, fatigue, muscle weakness, shortness of breath, muscle cramps Diarrhea: dark brown, odorless, low osmotic gap and secretory	Stool osmotic gap measurement Blood VIP cannot be measured in Japan
Somatostatinoma	Weight loss, abdominal pain, diabetes, cholelithiasis, steatorrhea, diarrhea, hypoacidity, anemia (often asymptomatic)	Blood somatostatin cannot be measured in Japan Diagnosis by biopsy
Carcinoid syndrome	Skin flushing (without sweating), diarrhea, pellagra symptoms, psychiatric symptoms (i.e., confusion), heart failure (especially right heart failure), bronchospasm, intra-abdominal fibrosis	Urinary 5-HIAA excretion measurement, intake of serotonin-containing foods and drugs

MEN1, multiple endocrine neoplasia type 1

Nonfunctional pancreatic NENs have no specific symptoms and may present with jaundice, pancreatitis, bloating, abdominal pain, or intestinal obstruction symptoms associated with tumor growth. Advanced cases are often identified owing to the detection of distant metastases [13]. Pathological diagnosis such as histology and cytology is recommended for differential diagnosis.

To determine the localization of pancreatic NENs, it is recommended to consider and perform imaging such as ultrasonography (US), computed tomography (CT), magnetic resonance imaging (MRI), endoscopic ultrasonography (EUS), or SRS on a case-by-case basis. When performing histology, endoscopic ultrasound-guided fine-needle aspiration (EUS-FNA) is recommended [3, 23]. If microscopic insulinomas and gastrinomas cannot be localized by imaging, then selective arterial secretagogue injection (SASI) test is useful [24, 25].

Endoscopic findings of gastrointestinal NETs are round, submucosal, tumor-like protrusions, which when grown are accompanied by central depression and ulceration [26]. Meanwhile, gastrointestinal NECs often appear as advanced cancer. The next recommended tests are endoscopic biopsy, EUS-FNA, and imaging to rule out distant metastases [27].

When testing for metastases, US, CT, MRI, positron emission tomography (PET) with 18F-fluorodeoxyglucose (FDG), or SRS should be performed as appropriate. For liver metastases, the rate of detection by US can be improved by the use of contrast media, and multiphasic imaging using contrast media is recommended for CT [28]. Contrast-enhanced MRI using Gd-DOTA yields higher detectability than CT or SRS [29]. Although only some well-differentiated NETs are positive on FDG-PET, FDG-PET is useful for finding metastases and recurrent lesions of tumors with high proliferative potential, such as NECs; this method is inversely correlated with and complementary to SRS. Although the sensitivity of SRS is not necessarily high at 52%, it has a high specificity of 93% [30].

## Histopathology

The WHO Classification of Endocrine Organs (2017) [5] and Digestive System (2019) [6] categorizes NENs as well-differentiated NENs (termed “NETs”) or poorly differentiated NECs. NENs are graded according to morphology (i.e., well or poorly differentiated) and proliferative activities (i.e., mitotic rate measured as mitoses/2 mm^2^ or Ki-67 index by counting 500 tumor cells in hotspots).

Well-differentiated NETs with proliferative activities < 3%, 3–20%, and > 20% are graded as G1, G2, and G3, respectively. In contrast, poorly differentiated NECs usually exhibit a higher Ki-67 index (i.e., > 20%) and lower expression of somatostatin receptor 2 (Table 2).

**Table 2:** WHO grading criteria for GEP-NENs (2017/2019)

Classification	Differentiation	Grade	Ki-67 index	Mitotic index (/2mm^2^)
NET G1 NET G2	Well differentiated	Low Intermediate	< 3% 3–20%	< 2 2–20
NET G3		High	> 20%	> 20
NEC				
Small-cell type Large-cell type	Poorly differentiated	High	> 20%	> 20
MiNEN	Well or poorly differentiated	Variable	Variable	Variable

GEP-NEN, gastroenteropancreatic neuroendocrine neoplasm; MiNEN, mixed neuroendocrine-–non-neuroendocrine neoplasm; NEC, neuroendocrine carcinoma; NET, neuroendocrine tumor

**Table 3:** Treatment approaches for gastroenteropancreatic neuroendocrine neoplasms

	NETs		NECs	
	Pancreatic origin	Gastrointestinal origin	Pancreatic origin	Gastrointestinal origin
Local therapy	Primary: resection	Primary: resection, endoscopic treatment	Resection ± adjuvant chemotherapy^a^	
	Metastasis: resection, RFA (for liver metastasis), TACE (for liver metastasis)	Metastasis: resection, RFA (for liver metastasis), TACE (for liver metastasis)		
Symptom management: somatostatin analogues	Octreotide		Octreotide	
	Lanreotide		Lanreotide	
Tumor control: somatostatin analogues	Lanreotide	Octreotide	–	–
		Lanreotide		
Tumor control: molecular targeted drugs	Everolimus	Everolimus	–	–
	Sunitinib			
Tumor control: cytotoxic anticancer agents	Streptozocin		Etoposide/cisplatin	
	Temozolomide^a^		Irinotecan/cisplatin	
	–		Etoposide/carboplatin	
Tumor control: radiation	Radiation (for bone metastases, brain metastases): PRRT*		Radiation (for bone or brain metastasis)	

^a^Off-label in Japan

In recent years, a high response rate of temozolomide therapy for pancreatic NET has been reported overseas [64]. Based on these results, guidelines also recommend temozolomide combination therapy is recommend as options for patients with large tumors and symptomatic patients in Europe and the U.S. Temozolomide combination therapy is one of the useful treatments, but it is not approved for insurance in Japan. Furthermore, radionuclide-labeling peptide therapy (PRRT) [65] is often used in Europe and the U.S. as well. PRRT has recently been covered by insurance in Japan, but at present, it should be given priority to patients who are ineffective with other drugs after the second treatment and need immediate PRRT treatment.

It can be difficult to distinguish NET G3 from NECs. However, the pathology of NET G3 is essentially similar to that of NET G1 and G2 in that it forms well-demarcated, medullary, expansive, solid masses; grows relatively slowly; and has a compact organoid structure (e.g., funicular, alveolar, pseudoglandular, etc.) with neuroendocrine differentiation on histology (Figure S1). Cytologic atypia remains mild to moderate, and components corresponding to NET G1 and G2 coexist inside the tumor. On the other hand, NECs form poorly demarcated medullary masses and grow rapidly. Histologically, highly atypical cells exhibit large alveolar to sheet-like and diffuse proliferation with an ill-defined organoid structure. NET G3 and NECs both have Ki-67 indices > 20%, but NECs usually have a Ki-67 index > 50% often with extensive necrotic foci; meanwhile, NET G3 rarely has a Ki-67 index > 50% or necrotic foci. Somatostatin receptor expression is often positive in NET G3 but weakly positive or negative in NECs. As NECs exhibit p53 overexpression and deletion of Rb protein, which are characteristic of extremely malignant tumors, immunostaining for these markers is helpful for differentiating NET G3 and NECs [3, 32, 34].

The WHO 2010 classification includes a category for mixed adeno-neuroendocrine carcinoma (MANEC) as combined adenocarcinoma and NET, although this did not include combinations such as acinar cell carcinoma and NET. In the new classification, such combinations are instead described as tumors involving both neuroendocrine and non-neuroendocrine cells, and are classified as mixed neuroendocrine–non-neuroendocrine neoplasm (MiNEN) [6].

## Surgical treatment

**Fig. 1:**
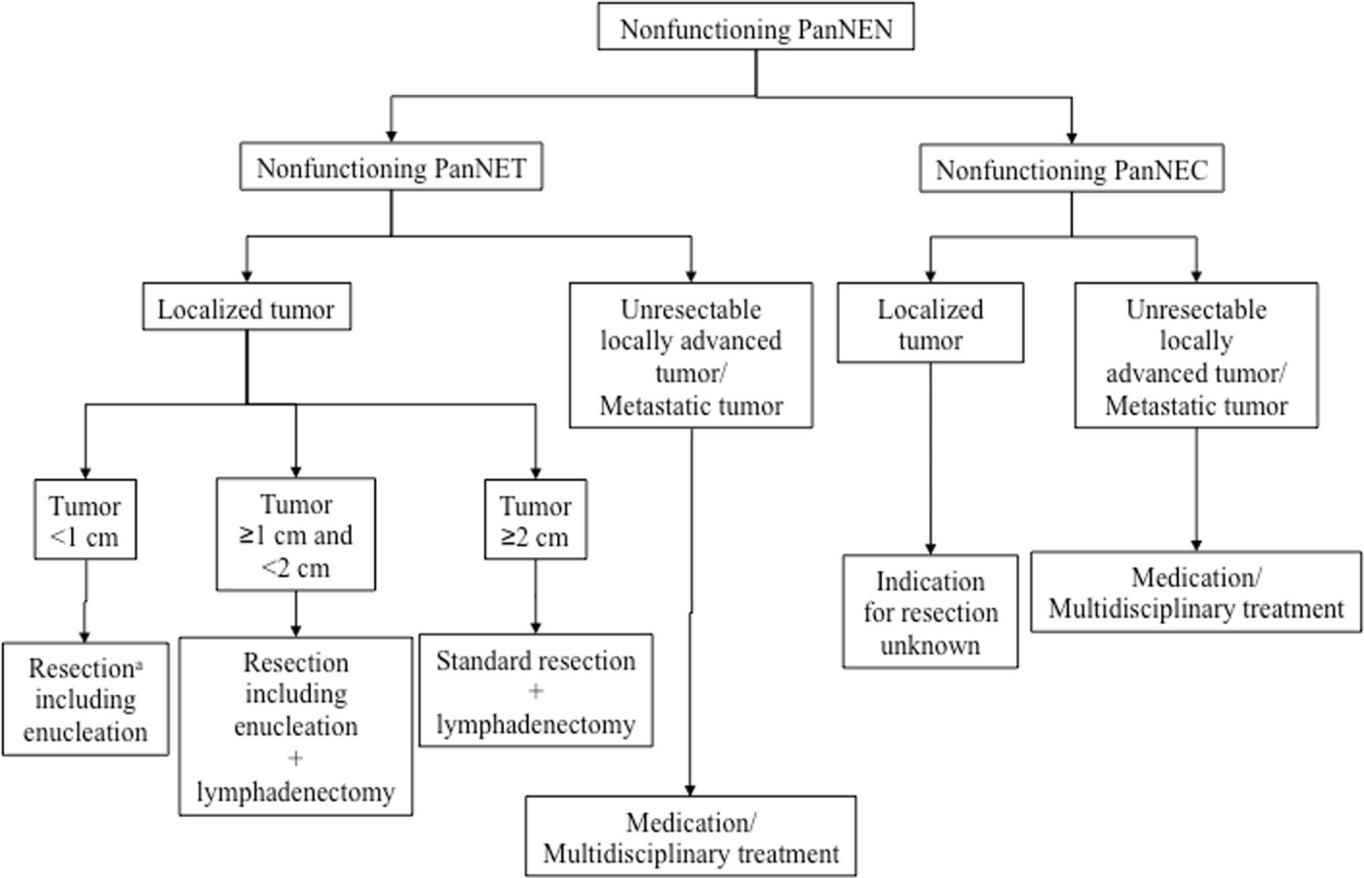
Surgical approach for nonfunctioning pancreatic NETs. (Superscript a) Check the swelling and firmness of the regional lymph nodes and dissect if lymph node metastases are suspected; if the tumor is discovered incidentally and there is no radiographic evidence of metastasis or invasion, follow-up may be an option with adequate explanation. PanNEC, pancreatic neuroendocrine carcinoma; PanNEN, pancreatic neuroendocrine neoplasm; PanNET, pancreatic neuroendocrine tumor

The surgical treatment of pancreatic NENs varies depending on the type of tumor. Pancreatectomy with lymphadenectomy is generally recommended for nonfunctioning pancreatic NENs [34, 35]. Meanwhile, for incidentally discovered asymptomatic tumors < 10 mm with no evidence of metastasis/invasion (e.g., hepatic or lymphatic involvement, pancreatic duct stenosis, and biliary stricture) on imaging, follow-up every 6–12 months may be an option with the patient’s informed consent (Fig. 1) [36–39]. Surgery is generally recommended for insulinomas; when indicated, a minimally invasive approach is preferable [40, 41]. Pancreatectomy with lymphadenectomy is recommended for malignant insulinomas [42]. For gastrinomas, the high malignant potential is assumed, and resection with lymphadenectomy is recommended. Gastrinomas with MEN1 exhibit metachronous recurrences, so care must be taken to avoid excessive surgery [43]. As rare functional pancreatic NENs other than insulinomas and gastrinomas (e.g., glucagonomas, VIPomas, somatostatinomas, GRFomas, PPomas, ACTHomas, and PTHomas) are highly malignant, pancreatectomy with lymphadenectomy is recommended [44].

When feasible, macroscopic curative resection is recommended for pancreatic NET G3 as for NET G1 and G2, whereas the indications for surgery are unclear for pancreatic NECs [45].

**Fig. 2:**
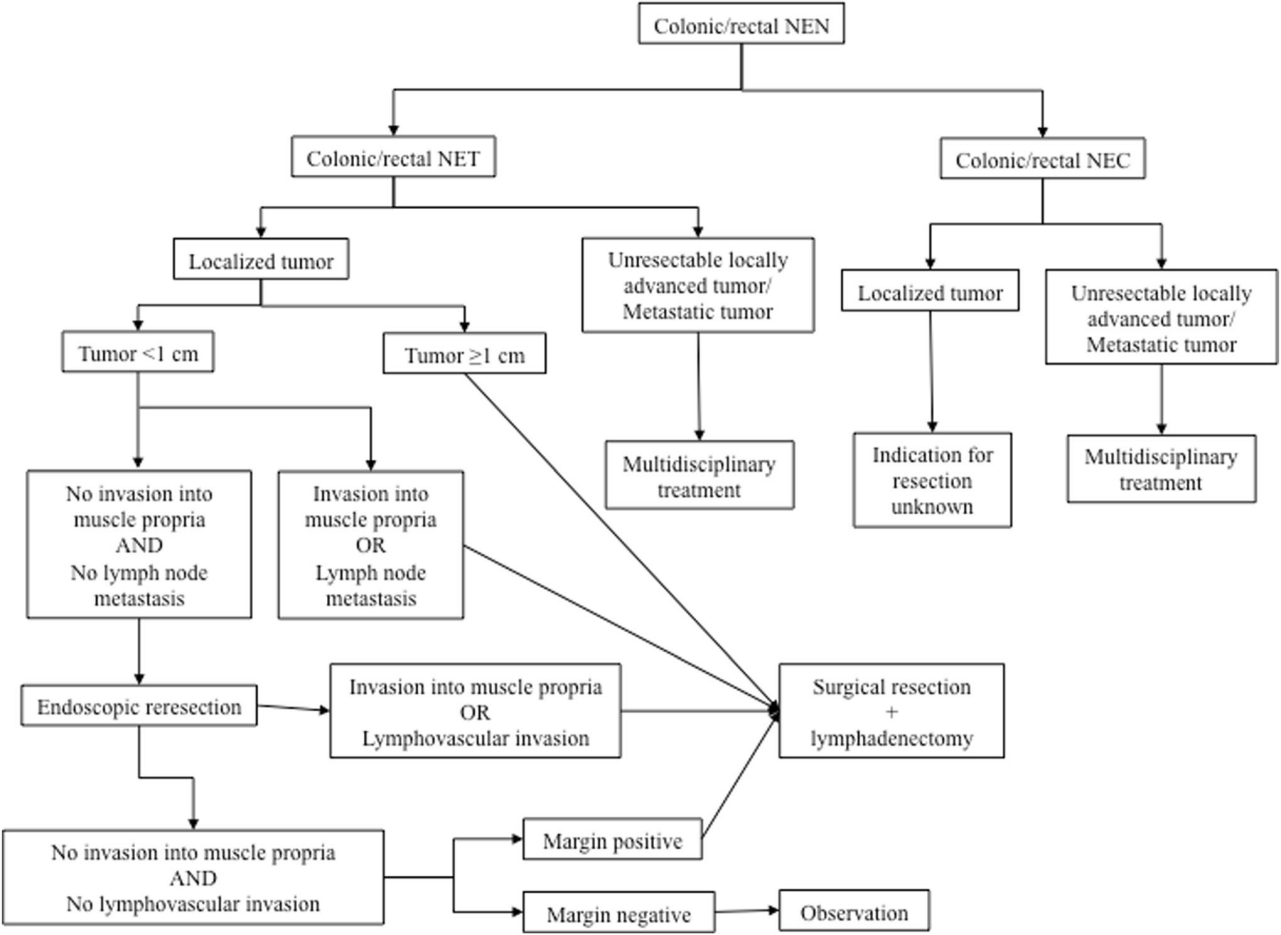
Surgical approach for NENs of the colon and rectum. NEC, neuroendocrine carcinoma; NEN, neuroendocrine neoplasm; NET, neuroendocrine tumor

Surgical treatment of gastrointestinal NENs varies by organ. More than 90% of esophageal NENs are NECs. Endoscopic resection or surgical resection is indicated for NETs according to stage, and drug therapy is indicated for nonresectable cases. Esophageal NECs are frequently accompanied by lymph node metastases (30%) and distant metastases (50%), requiring more careful decisions on surgery than that for esophageal cancers [46, 47]. For gastric NENs, the decision on surgical indications and selection of a surgical procedure according to Rindi’s classification [48] is recommended. For small intestinal NENs, small intestine resection with lymphadenectomy is recommended when curative resection is feasible [49]. Surgery is indicated for all appendiceal NENs, and the surgical approach should be selected by taking tumor localization, tumor size, and the presence of risk factors into account [50, 51]. Endoscopic resection is often indicated for colonic NENs, but colectomy with lymphadenectomy is recommended for the following: tumor size ≥ 1 cm or G2 or higher; muscularis propria invasion; suspected lymph node metastasis; or endoscopic resection specimens indicative of vascular invasion, muscularis propria invasion, positive surgical margins, or G2 or higher. Proctectomy with lymphadenectomy or rectal amputation is recommended for rectal NENs for the following: tumor size ≥ 1 cm or G2 or higher; muscularis propria invasion; suspected local lymph node involvement; or endoscopic resection specimens indicative of the need for additional treatment (Fig. 2) [52–54].

## Medical and multidisciplinary treatment (Fig. 3)

**Fig. 3:**
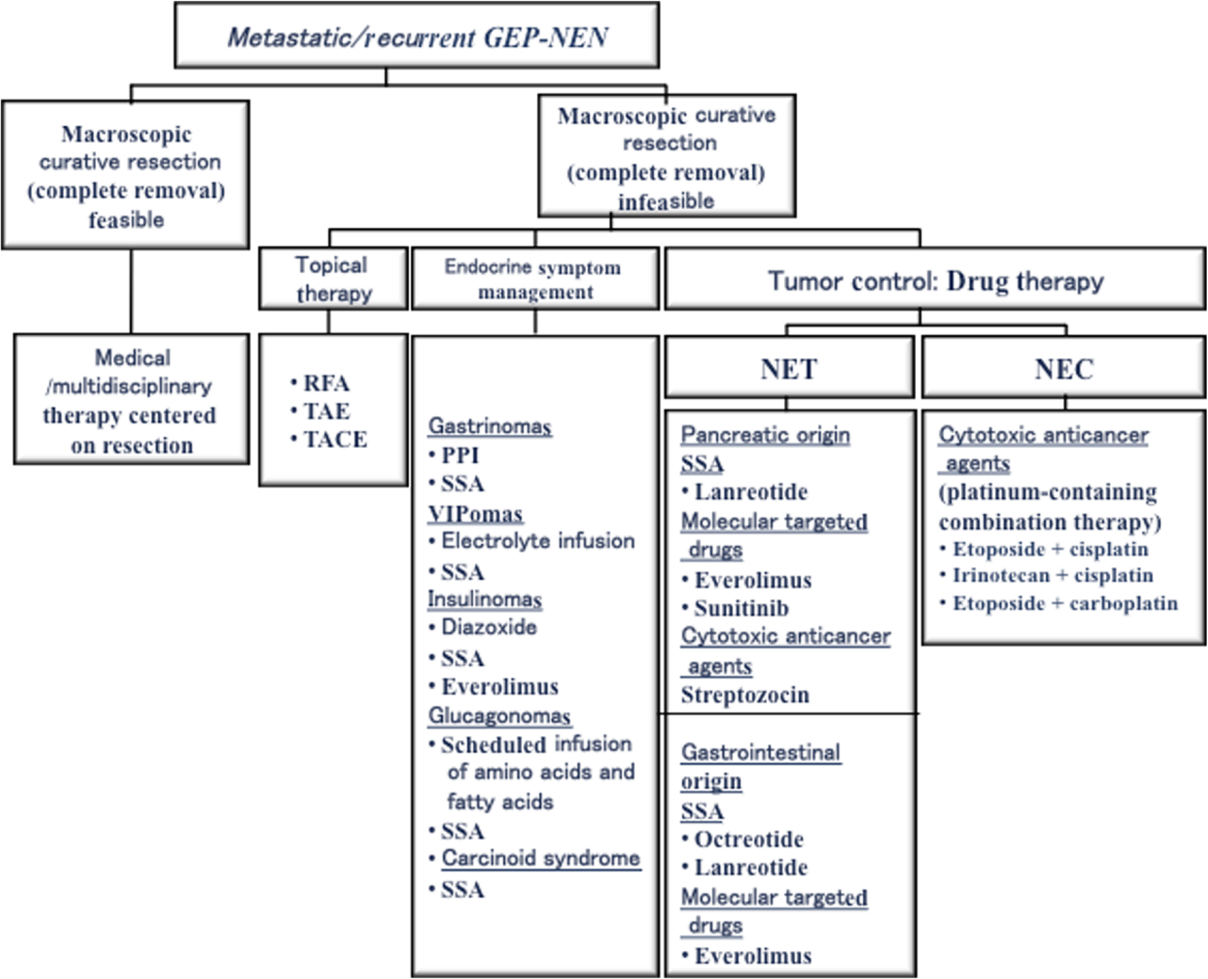
Treatment strategy for metastatic/recurrent GEP-NEN. GEP-NEN: gastroenteropancreatic neuroendocrine neoplasm; NEC: neuroendocrine carcinoma; NET: neuroendocrine tumor; PPI: proton pump inhibitor; RFA: radiofrequency ablation; SSA: somatostatin analogue; TACE: transarterial chemoembolization; TAE: transcatheter arterial embolization

Treatment modalities for GEP-NENs differ for NETs and NECs, and treatment approaches differ for NETs originating in the pancreas and gastrointestinal tract (Table 3). Resection is indicated for NETs when feasible; endoscopic treatment can also be considered for gastrointestinal NETs. Moreover, radiofrequency ablation and transarterial chemoembolization are used for liver metastases. However, adjuvant chemotherapy to prevent recurrence of NETs has not been established [55–57]. For functional NETs with hormonal symptoms, somatostatin analogues such as octreotide and lanreotide are used to control symptoms [55–58]. For tumor control, somatostatin analogues, molecular targeted drugs, and cytotoxic anticancer agents are indicated [55–57]. Regarding somatostatin analogues, insurance covers lanreotide [8] for pancreatic NETs as well as octreotide [59] and lanreotide [8] for gastrointestinal NETs. Regarding molecular targeted drugs, insurance covers everolimus [60] and sunitinib [61] for pancreatic NETs as well as everolimus [7] for gastrointestinal NETs. Regarding cytotoxic anticancer agents, insurance covers streptozocin [62, 63] for both pancreatic and gastrointestinal NETs; in addition, temozolomide [64] is considered promising in Europe and the U.S. Temozolomide combination therapy is one of the useful treatments, but it is not approved for insurance in Japan. Furthermore, radiation therapy may be used for palliative purposes for bone and brain metastases. Moreover, radionuclide-labeling peptide therapy (PRRT) [65] is often used in Europe and the U.S. PRRT has recently been covered by insurance in Japan, but at present, it should be given priority to patients who are ineffective with other drugs after the second treatment and need immediate PRRT treatment. For that purpose, it is considered necessary to build a network with feasible facilities.

While there is no established strategy for selecting appropriate treatment modalities, Japanese experts have proposed guidelines for NETs originating in the pancreas [66], although future validation is required.

For NECs, resection is indicated when feasible, and adjuvant chemotherapy can be used to prevent recurrence after surgery. Resection is not recommended for liver metastases in NECs. In nonresectable cases, platinum-based chemotherapy is indicated, such as etoposide/cisplatin, irinotecan/cisplatin, and etoposide/carboplatin [1–3]. However, no effective drug therapy has been established for cases refractory to these therapies [1–3].

Although GEP-NENs are rare, there are effective local therapies such as resection, radiofrequency ablation, and transarterial chemoembolization [55–57]. Several randomized controlled trials have demonstrated the usefulness of various drugs—many of which have been approved for use. In practice, the multidisciplinary treatment that takes advantage of these therapies is offered.

## MEN1/VHL disease

Some pancreatic NENs develop in settings of hereditary neoplasms, specifically MEN1 and VHL disease. Epidemiological studies report the frequencies of these hereditary neoplasms among all pancreatic NENs, and whole-genomic sequencing revealed that approximately 6% and 1% of pancreatic NENs carry germline mutations in MEN1 and VHL, respectively [67], suggesting that the actual frequency is within this range.

Pancreatic NENs in settings of hereditary neoplasms require different treatment approaches and surveillance compared to nonhereditary cases and also lead to preclinical diagnosis in family members. Therefore, it is important to appropriately screen patients with suspected hereditary neoplasia. In settings of MEN1, GEP-NEN is an important prognostic factor along with thymic NEN [68, 69]. Meanwhile, VHL disease rarely affects prognosis.

Regarding diagnosis, MEN1 or VHL disease is suspected in cases of GEP-NENs that meet the criteria in Table 4, thus requiring further assessment including searching for associated pathologies and genetic testing.

**Table 4:** Criteria for suspected MEN1 or VHL disease in cases of gastroenteropancreatic neuroendocrine neoplasms

1. Multiple pancreatic NETs 2. Recurrent pancreatic NETs 3. Gastrinomas (particularly of duodenal origin) (NEN-1) 4. Insulinomas in younger patients (MEN-1) 5. Complicated by hypercalcemia (MEN-1) 6. Presence and history of MEN1 or VHL-associated neoplasms
7. Familial history of MEN1 or VHL-associated neoplasms

MEN1, multiple endocrine neoplasia type 1; NET, neuroendocrine tumor; VHL, von Hippel–Lindau

MEN1 or VHL disease may also be associated with multiple small pancreatic NENs, and EUS-FNA with CT or MRI is recommended for localization. If a functional tumor is suspected, other nonfunctioning tumors are also often present; therefore, a SASI test is recommended [70].

The indications for surgery for GEP-NENs in settings of MEN1 or VHL disease are essentially the same as those for sporadic cases. However, because they involve multifocal and recurrent tumors, follow-up is generally recommended for nonfunctioning tumors < 2 cm in a setting of MEN1 [71]. Surgery is considered for tumors that are ≥ 2 cm or have a high growth rate. If surgery is indicated, a procedure that preserves as much pancreatic function as possible is recommended. In a setting of VHL disease, surgery is considered for tumors that are ≥ 2 cm and have a doubling time < 500 days [72].

Regarding surveillance, the growth rate of GEP-NETs associated with MEN1 is slow (0.1–1.3 mm/year); tumors < 1 cm, in particular, show little growth [73]. In patients with MEN1, annual follow-up including examination, imaging with CT or MRI, and biochemistry (i.e., fasting glucose, insulin, and gastrin) is recommended, keeping functional NENs in mind [74]. In patients with VHL disease, follow-up with dynamic CT every 2–3 years is recommended for tumors that are < 2 cm and have a doubling time ≥ 500 days and every 6 months to 1 year for tumors meeting only 1 of the 2 conditions.

## Conclusions

The revised clinical practice guidelines encompass the revised WHO classification for new diagnostic modalities, pathological diagnosis, surgical and medical/multidisciplinary treatments for pancreatic NETs, and the management of pancreatic NETs in a setting of hereditary diseases by addressing issues encountered in daily clinical practice.

Compared to countries other than Japan, the frequency of rectal NETs among gastrointestinal NETs is high in Japan, whereas the frequency of midgut NETs is high in Europe and the U.S. In addition, the frequency of MEN1 in cases of pancreatic NET is lower in Japan than that in Europe and the U.S. Accordingly, diagnostic and treatment approaches differ between patients from Japan and Europe or the U.S., requiring specific guidelines for patients in Japan. Thus, the revised guidelines contain specific strategies for GEP-NEN care in Japan, emphasizing clinical practicality.

### Supplementary Information

Below is the link to the electronic supplementary material.

Supplementary file1 (TIFF 1521 kb)

## Appendix

Members of the Guidelines Committee who created and evaluated the JNETS ‘‘Clinical practice guidelines for Gastroenteropancreatic Neuroendocrine Neoplasms’’ are listed below.

Executive Committee: Chair: Tetsuhide ITO (Neuroendocrine Tumor Centre, Fukuoka Sanno Hospital.

Department of Gastroenterology, Graduate School of Medical Sciences, Internal University of Health and Welfare).

Vice-Chair: Toshihiko Masui (Department of Surgery, Graduate School of Medicine, Kyoto University), and Izumi Komoto (Neuroendorine Tumor Center, Kansai Electric Power Hospital).

Members: Ryuichiro Doi (Department of Surgery, Otsu Red Cross Shiga Hospital), Robert Y. Osamura (Department of Diagnostic Pathology Nippon Koukan Hospital), Akihiro Sakurai (Department of Medical Genetics and Genomics, Sapporo Medical University School of Medicine), Masafumi Ikeda (Department of Hepatobiliary and Pancreatic Oncology, National Cancer Center Hospital East), Koji Takano (Department of Endocrinology, Diabetes and Metabolism, Kitasato University School of Medicine), Hisato Igarashi (Igarashi Medical Clinic), Akira Shimatsu (Advanced Medical Care Center, Kusatsu General Hospital), Kazuhiko Nakamura (Department of Gastroenterology and Hepatology, National Hospital Organization Fukuokahigashi Medical Center), Yuji Nakamoto (Department of Diagnostic Imaging and Nuclear Medicine, Graduate School of Medicine Kyoto University), Susumu Hijioka (Department of Hepatobiliary and Pancreatic Oncology, National Cancer Center Hospital), Koji Morita(Division of Endocrinology and Metabolism, Department of Internal Medicine, Teikyo University School of Medicine), Yuichi Ishikawa (Department of Pathology, School of Medicine, International University of Health and Welfare), Nobuyuki Ohike (Division of Pathology, Shizuoka Cancer Center), Atsuko Kasajima (Department of Pathology, Technical University Munich), Ryoji Kushima (Department of Pathology, Shiga University of Medical Science), Motohiro Kojima (Division of Pathology, Exploratory Oncology Research and Clinical Trial Center, National Cancer Center), Hironobu Sasano (Department of Pathology, Tohoku University School of Medicine), Satoshi Hirano (Department of Gastroenterological Surgery II, Hokkaido University Faculty of Medicine), Nobumasa Mizuno (Department of Gastroenterology, Aichi Cancer Center Hospital), Taku Aoki (Second Department of Surgery, Dokkyo Medical University), Takeshi Aoki (Department of Surgery, Tohoku University Graduate School of Medicine), Takao Ohtsuka (Department of Digestive Surgery, Breast and Thyroid Surgery, Graduate School of Medical Sciences, Kagoshima University), Tomoyuki Okumura (Department of Surgery and Science, Faculty of Medicine, Academic Assembly, University of Toyama), Yasutoshi Kimura (Department of Surgery, Surgical Oncology and Science, Sapporo Medical University), Atsushi Kudo (Department of Hepatobiliary and Pancreatic Surgery, Graduate School of Medicine, Tokyo Medical and Dental University), Tsuyoshi Konishi (Cancer Institute Hospital of the Japanese Foundation for Cancer Research), Ippei Matsumoto (Department of Surgery, Kindai University Faculty of Medicine), Noritoshi Kobayashi (Department of Oncology, Yokohama City University Graduate School of Medicine), Nao Fujimori (Department of Medicine and Bioregulatory Science, Graduate School of Medical Sciences, Kyushu University), Yoshitaka Honma (Department of Head and Neck, Esophageal Medical Oncology, National Cancer Center Hospital), Chigusa Morizane (Department of Hepatobiliary and Pancreatic Oncology, National Cancer Center Hospital), Shinya Uchino (Noguchi Thyroid Clinic and Hospital Foundation), Kiyomi Horiuchi (Department of Breast and Endocrine Surgery, Tokyo Women's Medical University), Masanori Yamasaki (Division of Diabetes, Endocrinology and Metabolism, Department of Internal Medicine, Shinshu University School of Medicine).

Evaluation Committee: Chair: Masayuki Imamura (Neuroendorine Tumor Center, Kansai Electric Power Hospital).

Members: Koichi Hirata (Department of Surgery, JR Sapporo Hospital), Takuji Okusaka (Department of Hepatobiliary and Pancreatic Oncology, National Cancer Center Hospital), Mitsuhiro Kida (Department of Gastroenterology, Kitasato University School of Medicine), Wataru Kimura (Toto Kasukabe Hospital), Masao Tanaka (Department of Surgery, Shimonoseki City Hospital), Yoshiyuki Majima (NPO PanCAN Japan).

Main Collaborators: Jun Matsubayashi (Department of Surgery, Otsu Red Cross Shiga Hospital), Yuichi Sato (Nagaoka central general Hospital), Masau Sekiguchi (Cancer Screening Center/Endoscopy Division, National Cancer Center Hospital), Shinichi Abe (Japan Medical Library Association).

Observer: Robert T. Jensen (Cell Biology Section, Digestive Diseases Branch, National Institutes of Diabetes, Digestive and Kidney Diseases. National Institutes of Health, USA.).

The Japanese Neuroendocrine Tumor Society: President, Shinji Uemoto (Department of Hepato-Biliary-Pancreatic Surgery and Transplantation, Graduate School of Medicine, Kyoto University).
