# Supplementary figures and images for: JNETS clinical practice guidelines for gastroenteropancreatic neuroendocrine neoplasms: diagnosis, treatment, and follow-up: a synopsis
Source: J Gastroenterol. 2021 Sep 29;56(11):1033–44. doi: 10.1007/s00535-021-01827-7 (PMC8531106; doi:10.1007/s00535-021-01827-7)

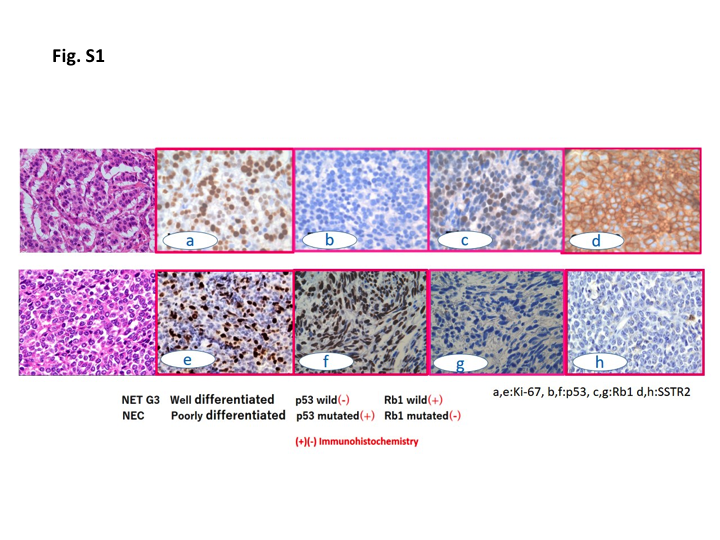

Supplement: Supplementary file 1 — Supplementary file1 (TIFF 1521 kb) [file 535_2021_1827_MOESM1_ESM.tiff]
